# Plasma-based optical fiber tapering rig

**DOI:** 10.1016/j.ohx.2024.e00578

**Published:** 2024-08-29

**Authors:** L.F. Granados-Zambrano, J.P. Korterik, J.M. Estudillo-Ayala, R. Rojas Laguna, D. Jauregui-Vazquez, H.L. Offerhaus, J.A. Alvarez-Chavez

**Affiliations:** a Optical Sciences Group – University of Twente, Drienerlolaan 5, 7522 NB Enschede, the Netherlands; b División de Ingenierías Campus Irapuato – Salamanca, Universidad de Guanajuato, Carretera Salamanca-Valle de Santiago km 3.5 + 1.8 km, Salamanca, Guanajuato 36885, Mexico; c Centro de Investigación Científica y de Educación Superior de Ensenada (CICESE), División de Física Aplicada-Departamento de Óptica, Carretera Ensenada-Tijuana, No. 3918, Zona Playitas, Ensenada 22860, Mexico

**Keywords:** Taper fabrication, Plasma, Arduino control, Thinned optical fiber sensors

## Abstract

Optical fiber tapers have been widely proposed and demonstrated as reliable optical fiber structures for sensing, lasers, and supercontinuum generation applications. This paper proposes an innovative approach to fabricating optical fiber tapers using plasma as the heat source. From our literature review, and to the best of our knowledge, this is the first time that plasma has been used as the heat source for producing optical fiber tapers. The system is not intricate and simple to replicate. Moreover, the elements involved make this machine attractive to research groups devoted to optical fibers. The setup consistently generates robust biconical optical fiber tapers. A typical waist of ∼8 μm and taper lengths ranging from 3 to 15 mm are achieved. Our results showed tapers with interference fringes up to 12 dB, from 1465 nm to 1599 nm. Furthermore, the statistical evaluation presented demonstrates a good level of reproducibility in our tapering process.

## Graphical abstract

**Figure:**
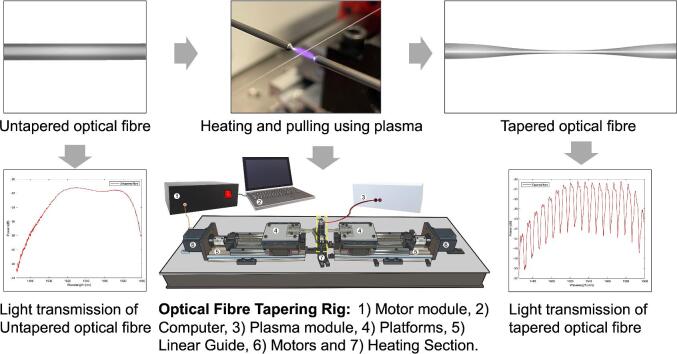

**Specifications table:** 

Hardware name	Plasma-Based Optical Fiber Tapering Rig
Subject area	• Opto-mechanical systems • Electronics • Optics • Engineering
Hardware type	• Measuring physical properties and in-lab sensors • Field measurements and sensors • Electrical engineering and computer science • Mechanical engineering
Open-source license	GNU General Public License (GPL)
Cost of hardware	555.94 − EUR
Source file repository	https://doi.org/10.17605/OSF.IO/VUW4J

## 1 Hardware in context

**Fig. 1:**
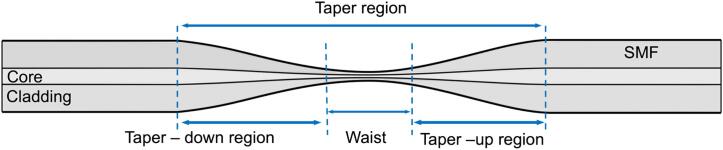
Schematic illustration of the tapered fiber.

The tapering process reduces the diameter of a single-mode fiber (SMF) over a determined length, as illustrated in Fig. 1, where the narrowest region is called the waist. The taper transitions are the regions where the diameter of the core and cladding are reduced. As the optical wave propagates through the transition region, the field distribution, or mode, changes and potentially mixes with other higher order modes, as a response to the changing core and cladding diameters. Consequently, the fundamental mode from the core in the untapered fiber, extends into the cladding and beyond in the taper waist region. Air, or other surrounding medium, is the new cladding, for light propagation at the cladding-air interface and the light that was confined in the fiber can interact with such medium.

The mode mixing and the interaction of the light with the surrounding medium allows for the measurement of a wide choice of physical and chemical parameters, such as strain and curvature [1], simultaneous refractive index and temperature [2], [3], gas detection [4], biosensing [5], among others. These capabilities make tapered fibers ideal for implementation in optical sensors. Moreover, these structures find applications in optical fiber lasers [6], supercontinuum generation [7], [8] and optical amplifiers [9], demonstrating their versatility in the advancement of optical technologies.

The proposed system provides a significant advantage compared to the expensive devices available. As mentioned earlier, it is important to note that, to the best of our knowledge, this is the first open-access high-precision manufacturing optical fiber tapers rig that uses plasma as fiber heating source; all the elements involved are commercially available and are composed of affordable hardware to build a simple electro-mechanical rig. Moreover, the plasma heating technique offers a novel, clean and controllable process. The system produces biconical structure tapers with a sound level of repeatability. This setup is controlled by an Arduino, allowing for customization, adaptation to other programming languages, and external hardware compatibility. The operational instructions are conveniently provided via the user interface of the Arduino IDE desktop application, offering a user-friendly and effort-less operating experience to fabricate the desired biconical optical fiber taper dimensions.

## 2 Hardware description

**Fig. 2:**
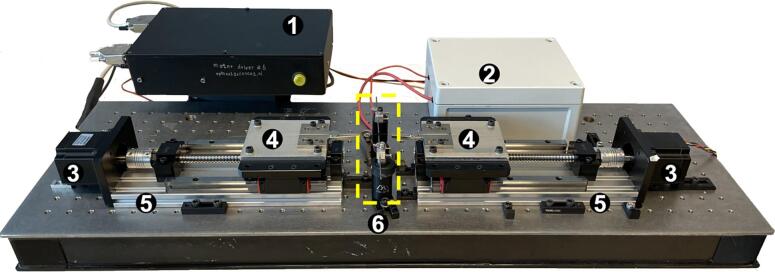
Plasma tapering Rig: 1) Motor control module, 2) Zero-Voltage Switching (ZVS) plasma module, 3) Stepper motors, 4) V-groove fiber mounts, 5) Motorized linear translation stages, and 6) Heating section.

The system consists of a mechanical configuration controlled by a circuit that receives operational instructions through a computer, resulting in a semi-automatic optical fiber tapering rig (Fig. 2).

**Fig. 3:**
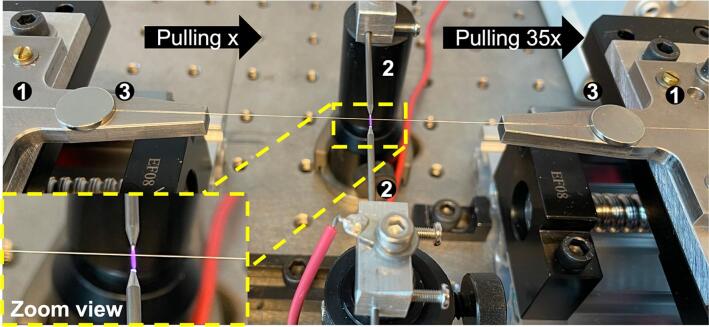
Tapering process showing the direction of pull and zoom view of the fiber through the plasma, where 1) V-grooves, 2) Tungsten electrodes and 2) Neodymium magnets.

The operating principle is the well-known fused tapering process. It starts by pulling the fiber using two fiber mounts on ultra-low vibration translation stages. The translation stages move in the same direction, but the stage on the right moves at a speed 35 times faster than the stage on the left, to simultaneously pull and transport the fiber through the plasma beam (see Fig. 3). The linear movement of the stages is achieved by using high-precision standard stepper motors that rotate a ball screw, enabling bidirectional movement. The translation stages smoothly glide on a metal linear rail with a low friction coefficient, ensuring a stable motion. The movement of the motors allows the user to define the desired linear travel distance operated through the serial monitor interface of the Arduino IDE. Besides, by the utilization of the micro-stepper driver TB6600, it is possible to amplify the resolution of angular motor movement and increase the resolution of the linear distance in micrometer units.

**Fig. 4:**
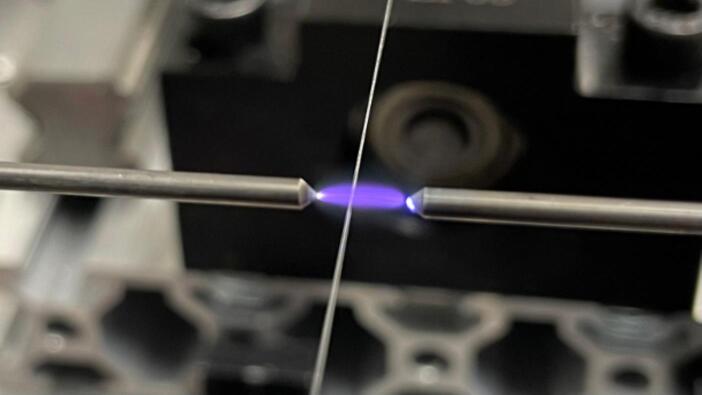
Heating section of the plasma acting on the bare fiber.

During the fiber pulling process, the ZVS Module using a Tesla coil generates plasma by inducing high voltage (>10 kV) which ionizes the air between the tungsten electrodes. The fiber is fixed with Neodymium magnets in the V-grooves, positioning the fiber between the tungsten electrodes. The heating region where the plasma is generated reaches temperatures exceeding 1200 °C (see Fig. 4). This heat softens the fiber, enabling the reduction of the diameter as it is drawn.

The length of the taper is determined by the travel distance of the faster translation stage. The waist diameter is a result of the interplay between two key factors: 1) The duration of exposure of the bare fiber to the plasma and 2) The rate at which the translation stages execute stretching. Plasma activation is established in the code, offering the flexibility for adjustments through simple programming modifications. The travel distance and speed are determined by the user according to the dimensions required. This approach allows for systematic exploration of parameters for taper fabrication, while demonstrating controlled reproducibility.

### 2.1 Comparison with some existing tapering fabrication rigs with different heating techniques

Several groups have developed their own tapering rigs. The main rigs with different heat-pulled techniques reported for splicing fiber are Flame brushing [10], [11], [12], CO2 Laser [13], [14], [15], Ceramic Microheater [16], [17] and Electric Arc Discharge [18], [19], [20]. Each technique has its own advantages and limitations, and the choice of technique depends on factors such as the fiber composition, the taper dimensions, adiabaticity and geometrical constraints, as determined by the application.

The rigs that use a CO_2_ laser or microheater show superior manufacturing performance, but require expensive technologies compared to plasma. The use of a combination of gases within a flame represents a cost-effective technique. However, flames are susceptible to fluctuations caused by turbulent gas flow and residual air currents within the environment. Also, contaminants present in the flame, such as soot particles, can adhere to the fiber surface during the tapering process. The Electric Arc Discharge and the plasma employ the same principle. They are less susceptible to contaminating silica. However, the setup with electric arc discharge requires higher power (>100 W) compared to plasma generation, leading to elevated temperatures (>20,000 °C), which are considered hazardous. Proper training and adherence to stringent safety precautions are paramount in such scenarios. Users should be equipped with appropriate personal protective equipment (PPE) to mitigate potential risks associated with elevated temperatures and intense light. Therefore, plasma combines the following advantages:

• Economical Implementation: plasma generation requires a commercially available, ultra-low-cost and safe circuit.
• Durable Electrodes: The proposed electrodes exhibit high resistance to the effects of discharges, resulting in prolonged durability.
• Safety and Simplicity: Unlike other methods, the plasma technique eliminates the necessity for flammable gases, enhancing overall safety while streamlining the operational procedure. Using a commercial flyback (see materials list) ensures safe operation through adherence to operational guidelines and safety protocols.
• Accessible Replication: The successful replication and operation of this method demands only fundamental/basic engineering knowledge, reducing barriers to replication and implementation.
• Efficiency in Taper Fabrication: The plasma technique boasts rapid manufacturing of tapers, aligning with the demands of time-sensitive applications.

While conducting this comparison, we investigated and contrasted the utilization of plasma as a new alternative to existing methods. The table below summarizes the results obtained using these techniques, according to literature (Table 1).

**Table 1:** Features of tapered fibers fabricated by different heating methods.

Tapering technique	Process time (min)	Waist diameter (µm)	Taper length (mm)	Insertion loss in air (dBm)	Reference
CO_2_ Laser	N/A	3 to 4	40	0.20	[13]
Ceramic microheater	4	∼0.25	40	0.20	[1]
Flame brushing	∼3	1	15	1.0	[11]
Electric arc	1	3	10	0.16	[18], [19]
Plasma	0.12	8	15	<1.5	**This work**

## 3 Design files summary

See Table 2.

**Table 2:** Design files summary.

Design file name	File type	Open source license	Location of the file
*Fiber Pulling Arduino Code*		GNU GPL v3	https://doi.org/10.17605/OSF.IO/VUW4J
Tapering process (Video)		CC BY 4.0	https://doi.org/10.17605/OSF.IO/VUW4J
Circuit diagram		CERN	https://doi.org/10.17605/OSF.IO/VUW4J

## 4 Bill of materials

### 4.1 Bill of materials summary

See Table 3.

**Table 3:** Bill of materials, the instructions for manufacturing the machined parts are found in the general file repository, with the same name as the part.

Designator	Component	Number	Cost per unit (Euro)	Total cost (Euro)	Source of materials	Material type
Arduino UNO	Arduino UNO Rev3	1	24.00	24.00	Arduino Uno Rev3—Arduino Official Store	Electronic
Stepper Motor Nema 23	NEMA23 HPV6 Linear module ball screw sfu1204 with Linear Guides HGH15 HIWIN with 2.8A 56 mm stepper motor	2	157.00	314.00	NEMA23 HPV6 Linear module ballscrew sfu1204 with Linear Guides HGH15 HIWIN 100 % same size with 2.8A 56 mm stepper motor − AliExpress	Hardware
Stepper Motors Driver	TB6600 Driver (4.0A / 9-42VDC)	2	10.00	20.00	https://www.amazon.com	Electronic
HBM Machines	Tungsten TIG welding electrode	2	1.70	3.40	HBM Wolfraam elektrode groen verpakt per 10 stuks | HBM Machines (hbm-machines.com)	Metal
Hilitand	Tesla Coil Driver Board ZVS Tesla Coil Flyback Driver Marx generator Tesla Coil Power Supply 12 to 36 V	1	20.00	20.00	Amazon.com: Tesla Coil Driver Board ZVS Tesla Coil Flyback Driver Marx generator Tesla Coil Power Supply 12 to 36 V: Automotive	Electronic
XP Power	AC/DC CONVERTER 12 V 36 W	2	12.65	25.3	LCS35US12 XP Power | Power Supplies − External/Internal (Off-Board) | DigiKey	Electronic
OTRONIC	5 V 2-Channel Relay interface board	1	6.00	6.00	OTRONIC® Relais Module 5v | 2-kanaals | Arduino | ESP32 | ESP8266 | Raspberry Pi | Wemos | bol.com	Electronic
−	3 pin connector AC 250 V 10A	1	3.00	3.00	Melitt IEC C14 C1 32pcs 3 pin chassis panel mount plug connector AC 250 V 10A black: Amazon.nl: Electronics & Photo.com)	Electronic
Magnet Expert	Neodymium Magnet	2	12.00	24.00	Ultra High Performance N52 Neodymium Magnet for Arts, Crafts, Model Making, DIY, Hobbies, Office, and Home − 15 mm dia × 2 mm thick − 2.4 kg Pull − Pack of 10: Amazon.co.uk: Business, Industry & Science	Metal
	V groove	2	3.00	6.00	Machined	Metal
	aluminum base 80 × 100 mm	2	3.00	6.00	Machined	Metal
	Aluminum holders (for electrodes)	2	3.00	6.00	Machined	Metal
−	KCD4 ON/Off Switch 4 Pin 16A/250 V	1	0.74	0.74	KCD4-201 on off on on 30A/250 V 16A/250 V heavy duty 4 pin t85 rocker switch with light 12 V 24 V 110 V 220 V 380 V − AliExpress	Electronic
	1set 4 mm Banana Plug	2	0.35	0.70	1set Male And Female 4 mm Banana Plug Male And Female To Insert Connector Banana Pin DIY Model Parts − AliExpress	Electronic
	D-Sub 15 female connector	2	2.0	4.0	https://www.amazon.nl/dp/B07LCLD96Y?psc=1&ref=ppx_yo2ov_dt_b_product_details	Electronic
	DC-099 Power Jack	1	1.8	1.8	https://www.amazon.com	Electronic
	Aluminum Enclosure, Black Lid, 275 × 175 × 65.5 mm	1	60.00	60.00	RS PRO Black Die Cast Aluminium Enclosure, Black Lid, 275 × 175 × 65.5 mm | RS (rs-online.com) − online.com)	Metal
	Plastic Enclosure 200 × 150 × 100 mm	1	31.00	31.00	Waterproof Plastic Enclosure Project Box Electronic IP67 Electrical ABS Junction | eBay	Plastic
Total				555.94 (EUR)		

## 5 Build instructions

The construction process consists of three procedures: the construction of the motor control module, the construction of the ZVS plasma Module, and the assembly of the mechanical setup.

### 5.1 Construction of the motor control module

In Fig. 6, we present a visual schematic that shows the electrical components and the wiring connections. Additionally, Fig. 7 provides the circuit diagram, with a detailed view of pin interconnections. The assembly instructions of the motor control module are described in the following procedure:

**Fig. 5:**
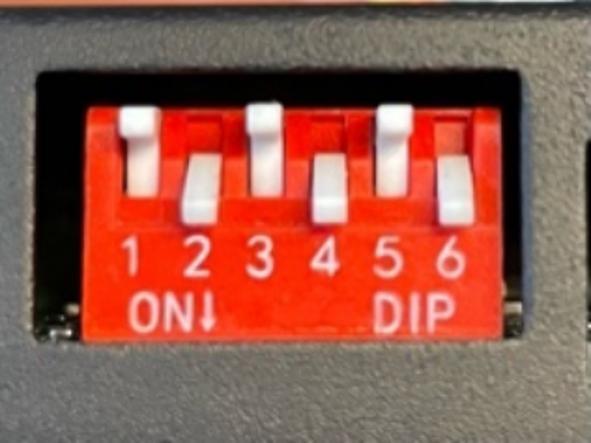
Configuration of the DIP switch of the Microstep Driver.

**Fig. 6:**
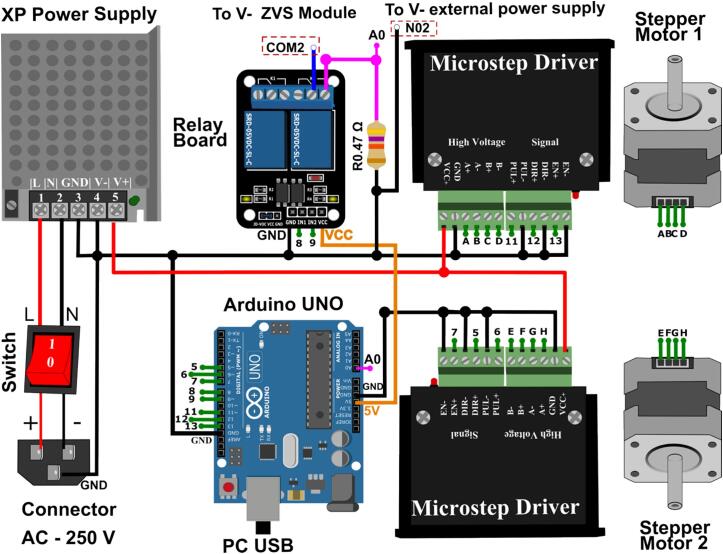
Motor control wiring diagram.

**Fig. 7:**
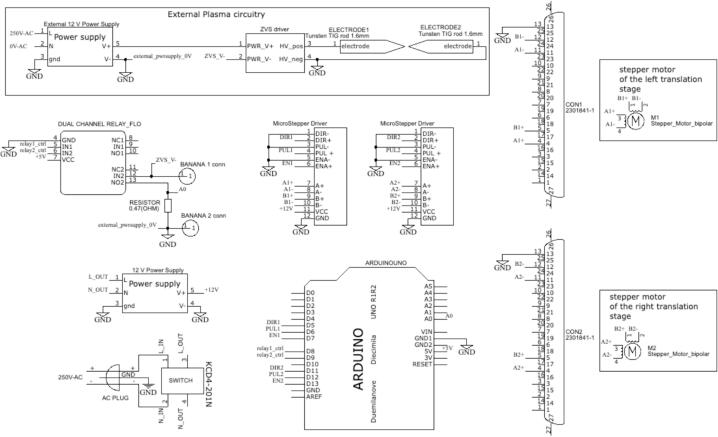
Circuit diagram of motor control and plasma modules.

1. Connect the AC connectors positive and negative pins to the inputs of the switch.

2. Link the output pins of the switch to the AC inputs of the XP Power Supply.

3. Establish a common ground connection as indicated by the black wire in the diagram. This step is crucial for the proper operation of the electrical circuit. Additionally, ensure that the aluminum enclosure and the optical table are also connected to the common ground.

4. Connect the power from the Microstep Driver to the 12 V output of the XP Power Supply, represented by the red wire.

5. Set the micro step via the DIP switch of the Microstep Driver, see Fig. 5.

The table below shows the ON/OFF configuration of the DIP switch, where the step angle setting for the motors is optimized at 0.225° (Table 4).

**Table 4:** DIP switch configuration.

SW1	SW2	SW3	SW4	SW5	SW6
OFF	ON	OFF	ON	OFF	ON

This configuration corresponds to 1600 pulses/revolution and an operational current of 2.5 A. Besides, for the linear rail track, each revolution corresponds to a linear movement of 4 mm.

(1) $$1600\;\text{steps}=1\;\text{rev}/\text{s}=4\left[\text{mm}\right]$$

Thus for 400 steps the travel distance corresponds to 1 mm

(2) $$400\;\text{steps}=1\left[\text{mm}\right]$$

As previously mentioned, both translation stages are moving to the right. Notably, the right stage travels at a speed and distance 35 times greater than the left stage. The lowest stage does not influence the determination of the taper length. This linear movement of the faster stages is represented by the following equation:

(3) $$\Delta D\;\left[\text{mm}\right]=\left(36x\right)D$$

where D represents the travel distance (1 mm per 400 steps), x denotes the number of steps, and AD represents the cumulative distance in millimeters.

1. Make the interconnections of the pins with the same label. Note that the COM2 and N02 pins of the relay control the on and off functions of the plasma module.

#### 5.1.1 Assembly of the motor circuit in the aluminum enclosure

For enclosure, the circuit in the aluminum enclosure is necessary to add and make the holes for the follow elements:

1. Switch
2. 2 Banana connectors
3. 2 D-sub 15 female connectors
4. 3 pin connector AC 250 V 10A
5. Hole for the cable USB B (Arduino PC Connection)

While users have the flexibility to position the components according to their preference, we recommend the following procedure for optimal connector placement.

1. Position the box horizontally and identify the faces indicated in Fig. 8

**Fig. 8:**
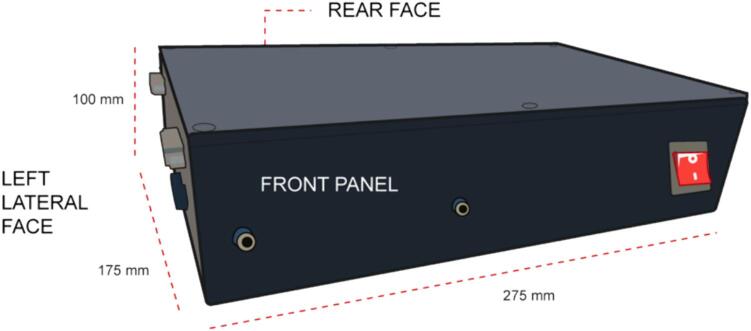
Aluminum enclosure illustration.
2. Located the front panel face and make the hole for the ignition switch, with the measures specified in Fig. 9.

**Fig. 9:**
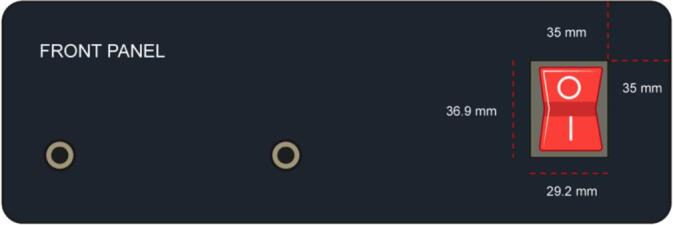
Front Panel face with the dimensions of the hole for the 1) switch.
3. In the lateral left face, make the openings for the installation of two banana connectors and two D-sub 25 connectors where the dimensions and position of the holes are specified in Fig. 10.

**Fig. 10:**
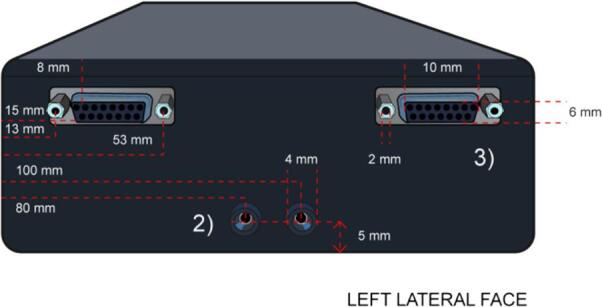
Left Lateral Face with the dimensions of the holes for the 2) Banana and 3) D-sub 15 connectors.
4. Lastly, create openings for the AC connector and the USB-B Arduino port on the rear face of the enclosure (see Fig. 11).

**Fig. 11:**
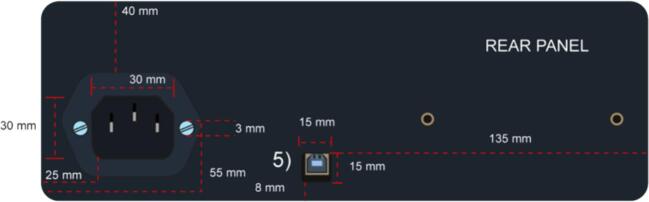
Rear Face with the dimensions of the holes for the 4) AC connector and 3) USB B (Arduino).
5. The components of the motor control module must be appropriately positioned within the aluminum enclosure, following the connections of the adapters mounted in the enclosure, as depicted in Fig. 12.

**Fig. 12:**
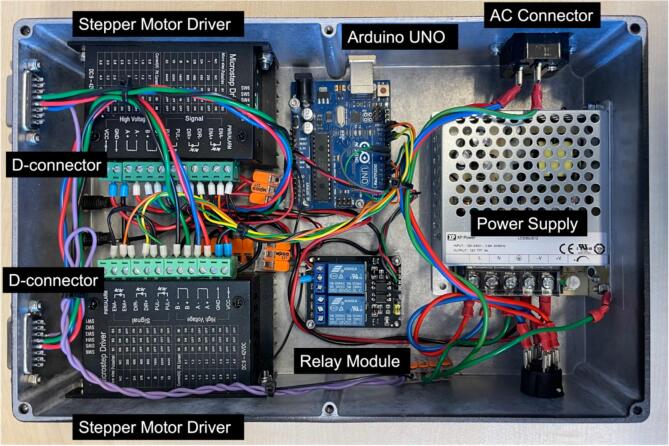
Internal view of the Motors control module.

### 5.2 Construction of plasma ZVS module

**Fig. 13:**
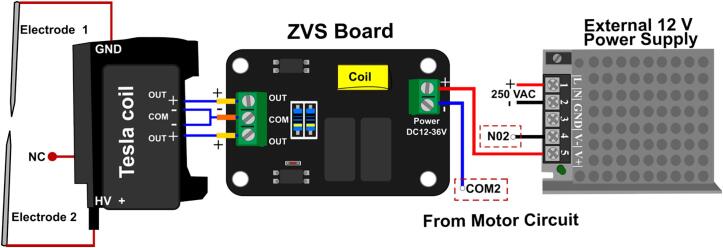
Plasma generator wiring diagram.

The plastic enclosure is necessary for the hardware of the plasma. Make a hole for the banana female plug corresponding to the 7 V external power supply. The external hardware wiring connections are depicted in Fig. 13, while Fig. 7 shows the circuit diagram of this module. The mounting instructions are describing in the following procedure:

1. Begin by establishing the connection between the motor control module and the plasma generator by setting the following configuration:
  • Connect the positive terminal of the external 7 V power source (+) directly to the input voltage terminal on the ZVS board.
  • Take the negative terminal of the external 7 V power source (−) and connect it to N02 on the motor circuit.
  • Finally, connect Pin COM2 of the motor circuit to the negative input terminal of the ZVS board.
2. Connect the blue wires from the coil to the power output of the ZVS controlled module. The orange-tipped paired wires must be connected to the central connector on the board, which corresponds to negative (−), while the other two yellow-tipped wires should be connected to the positive (+) ends of the connector.
3. Solder the two red ends of the Tesla coil red wires to the tungsten electrodes, where one is the high voltage positive electrode, and the other is ground.

Note that the ground wire must be connected to the ground of the AC mains that power the rig, and the other red wire located in the middle of the coil is not connected (NC).

#### 5.2.1 Assembly of the plasma circuit in the plastic enclosure

To mount the plasma generator in the plastic box, the elements must be positioned and secured at the bottom of the box. To install the connectors, the following steps must be performed:

1. Locate the smallest lateral faces, see Fig. 14.

**Fig. 14:**
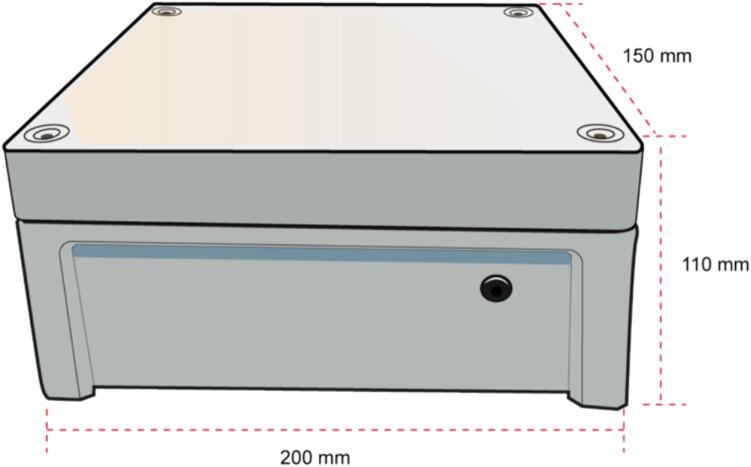
Plastic enclosure.
2. In the left face, make two holes for the two cables corresponding to high voltage and ground of the Tesla coil, the dimensions of the holes are shown in Fig. 15.

**Fig. 15:**
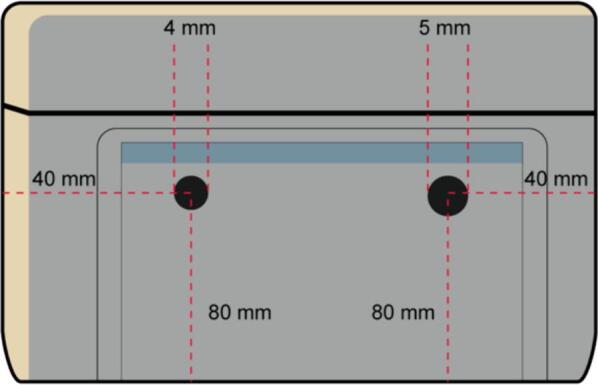
Left Lateral Face with the dimensions of the holes for the HV and GND cables.
3. Make the hole for the DC power adapter of the external power supply in the right face, with the dimensions described in Fig. 16.

**Fig. 16:**
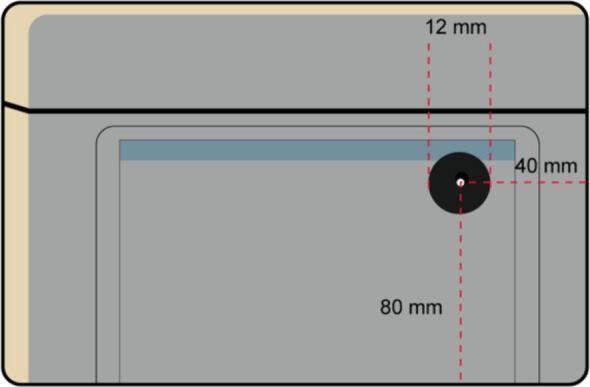
Right Lateral Face with the dimensions of the hole for the DC power adapter.
4. The plasma module is conveniently situated within the plastic enclosure. The High Voltage and Ground cables are connected to the tungsten electrodes, and the female DC power adapter serves as the interface for the external 12 V Power Supply, (see Fig. 17).

**Fig. 17:**
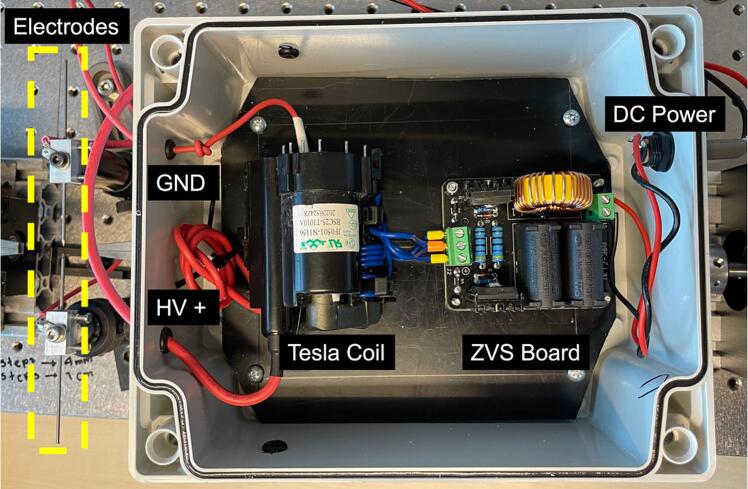
Internal view of the plasma module.

### 5.3 Assembly of the mechanical setup

To construct the mechanical setup, you will require a workspace of 0.3 square meters, which equates to 1000 × 300 × 60 mm (length × width × height) on a leveled surface. We recommend using an optical table to mount the experiment, as this will minimize motor-induced vibrations and enhance precision in distance measurements.

For improved clarity regarding the assembly process (refer to Fig. 2), please follow these steps:

1. Place the linear translations stages in a front-to-front orientation, maintaining a 100 mm gap between them (Fig. 18).

**Fig. 18:**
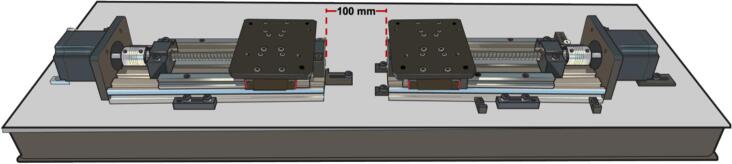
Illustration of linear guide module placement.
2. Position the linear translation stages with precision, ensuring symmetry on the worktable and perfect alignment with each other. Use the edges of the table as your reference point.
3. Fix the elements using clamp packs in case of optical table.
4. Collocate the aluminum base and the v-groove on the plates of the platforms with correct screws according to the diameter of the holes describing in the machined material figures or in the following Fig. 19.

**Fig. 19:**
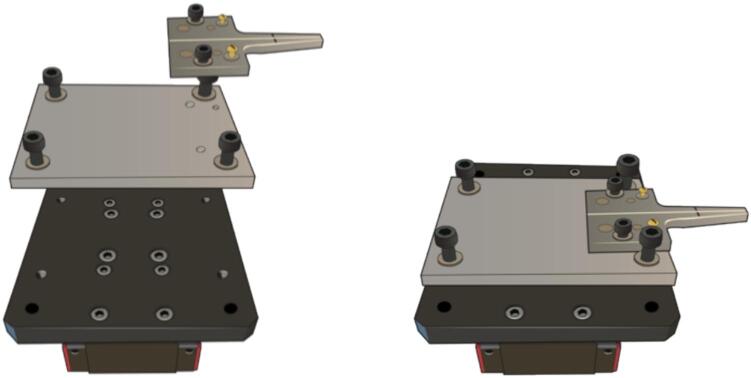
Illustration of base and v-groove assembly.
5. Introduce the tungsten electrodes into their machined holder, securing them in place with the corresponding screws, and mounting the assembly onto an optical post. The HV+ holder should have an isolated post with at least 20 mm thickness with respect to the optical table to avoid arcing. The ground post must be connected to the common ground of the electrical circuit and directly to the optical table (Fig. 20)

**Fig. 20:**
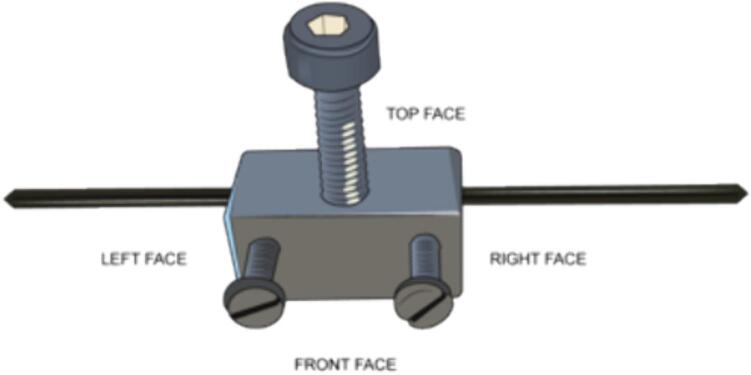
Tungsten electrode placed in machined holder.
6. Place the tungsten electrodes between the translation stages. Ensure that the electrodes are set at the same height in relation to the top of the V-groove. It is advisable to use optical posts for precise control during this step (Fig. 21).

**Fig. 21:**
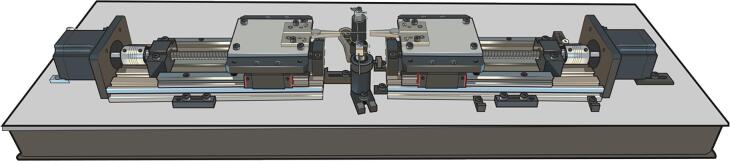
Illustration of electrodes placement.
7. Fix the electrodes with 6 mm distance between them (Fig. 22).

**Fig. 22:**
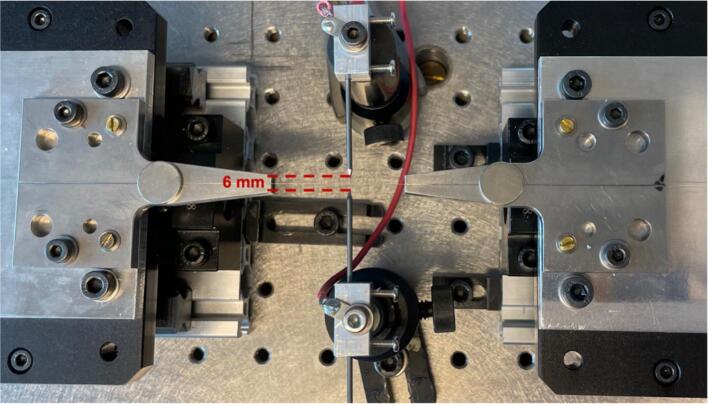
Photograph showing the placement of the electrodes between the V-grooves.
8. To guarantee that the space between the electrodes aligns perfectly with the V-grooves, employ optical fiber for the alignment process.
9. Finally, establish the electrical connections by connecting the motor cables to the motor control module and the electrodes to the Plasma ZVS Module.

## 6 Operating instructions

1. Install the Arduino IDE software (only the first time)
2. Connect the Arduino to the PC
3. Open the plasma control Arduino program
4. Upload the program to the Arduino (Be sure to select the Arduino UNO board and the right USB port of the PC)
5. Install the AccelStepper library by Mike McCauleys, version 1.64 directly from the Arduino IDE library manager. (Only once)
6. Turn on the Motors Control Module
7. Energize the plasma Module
8. Open the Serial Monitor in Arduino IDE
9. Fig. 23 illustrates the flow diagram of the taper manufacturing process. Please ensure close attention is paid to the next instructions, (see 'taper manufacturing video', attached in the repository).

**Fig. 23:**
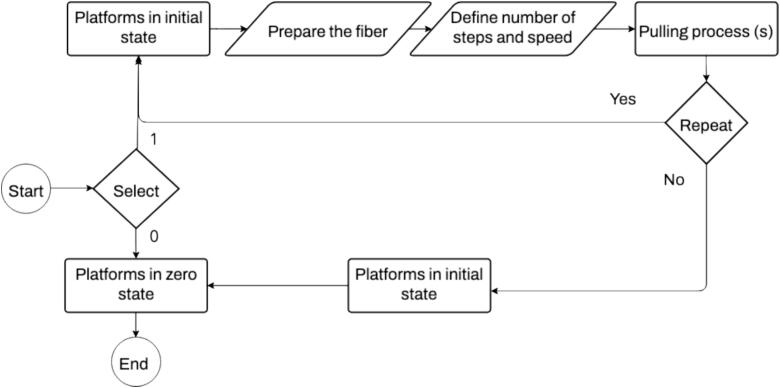
Flow diagram of pulling process.
10. Considering that the platforms are positioned manually at the end of the rail, enter the number '1′ and press enter to move the platforms to an initial state before pulling. While introducing '0′ the platforms moving in the home state or zero state.
11. When the platforms are in the initial state, prepare the fiber: remove the coating of the section of the fiber that will be thinner, it depends on the length of the taper that you want to fabricate. (see 'fiber preparation video', attached to the repository).
12. Place the bare fiber between the electrodes and fixed the fiber using the magnets in the V-groove, (see Fig. 3).
13. Determine the steps for the required length of the taper. This length is determined using Eq. (4), or the simplified form presented in Eq. (5), where 'x' represents the number of steps.

(4) $$\mathrm{Taper}\;Length\;\left[\text{mm}\right]=\frac{1\;\left[\text{mm}\right]}{400\;\left[\text{steps}\right]}\left(35x\right)\left[\text{steps}\right]$$

(5) $$\mathrm{Taper}\;Length\;\left[\text{mm}\right]=0.0875x\;\left[\text{mm}\right]$$

The diameter was established based on near-to-optimal performance observed during tests. After obtaining 10 µm with 15 steps/s, the user can explore at higher operational speed whereas it is possible to reduce the diameter of the taper.

The following table presents the dimensions of the taper, along with the fabrication time, based on pre-determined travel distance and selected speeds (Table 5).

14. For the pulling process, introduce the capital letter 'R' to indicate movement to the right. Immediately following the letter, input the number of steps, and with a space, introduce the speed (see Fig. 24). Press Enter to start the process.

**Fig. 24:**
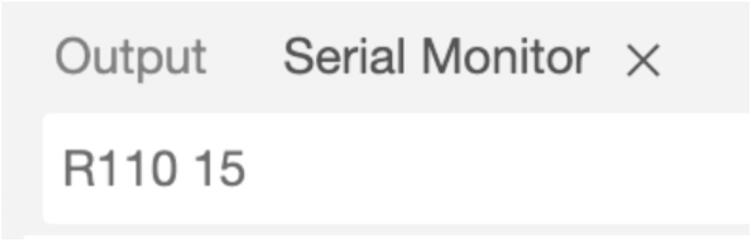
Serial Monitor instructions for pulling.
15. After each pulling process, it is necessary to return to the initial state with the same parameters introduced for pulling but changing the letter 'R' to 'L' (see Fig. 23). During this leftward movement, the plasma is not activated (Fig. 25).

**Fig. 25:**
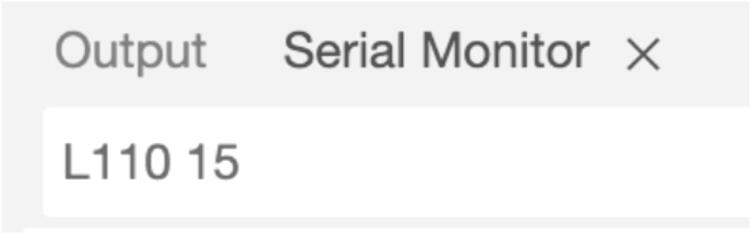
Serial Monitor instructions for return.
16. Carefully remove the tapered fiber
17. Upon completion of the process, position the platforms in the zero-state.

**Table 5:** Optical taper fabrication guidelines.

No. 1	Travel distance (steps)	Speed (steps/s)	Waist diameter (µm)	Length (mm)	Fabrication time (s)
1	55	15	8	5	3.6
2	110	15	8	10	7.3
3	165	15	6	15	11

## 7 Validation and characterization

### 7.1 The shape of the tapered structure

**Fig. 26:**
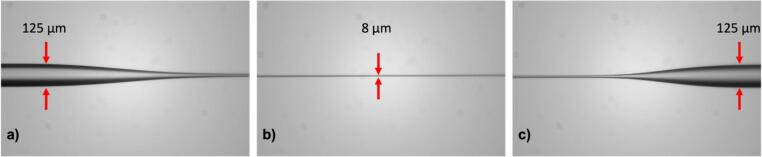
The actual image of a tapered fiber with a length of 10 mm and a diameter of 8 µm, depicting a) down tapered transition, b) waist, and c) up tapered transition.

**Fig. 27:**
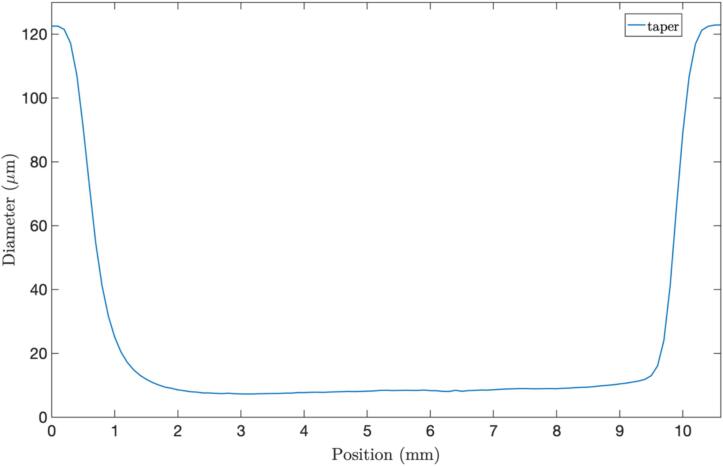
Fiber taper profile of a tapered fiber with a length of 10 mm and a diameter of 8 µm.

For characterizing the taper dimensions, we use a FLIR BFLY-U3-23S6M-C camera with 5.86 µm * 5.86 µm pixel unit cell size in conjunction with a 10× microscope objective and the 10 cm tube length this translates to a design resolution of ∼0.7 µm per pixel in the obtained images. Images of the tapers were captured to determine their dimensions and transition shapes (see Fig. 26). Employing image processing, we measured the position of the edges of the fiber. A picture was taken every 0.1 mm across the tapered region from which the fiber profile curve was determined, as illustrated in Fig. 27.

### 7.2 Fabrication statistics process

To demonstrate the level of control over the fabrication process we manufactured several tapers with the same settings and calculated the manufacturing process's statistics. In the following table, we present the average results of 10 tapers fabricated with 110 steps of travel distance and 15 steps/s, resulting in (Table 6):

**Table 6:** Statistical analysis of 10 fabricated tapers.

Number of tests	Temperature [°C]	Humidity [%]	Tapered fiber length [mm]	Waist length [mm]	Repeatability (%)
10	21.4	41	10	8	7.3

**Fig. 28:**
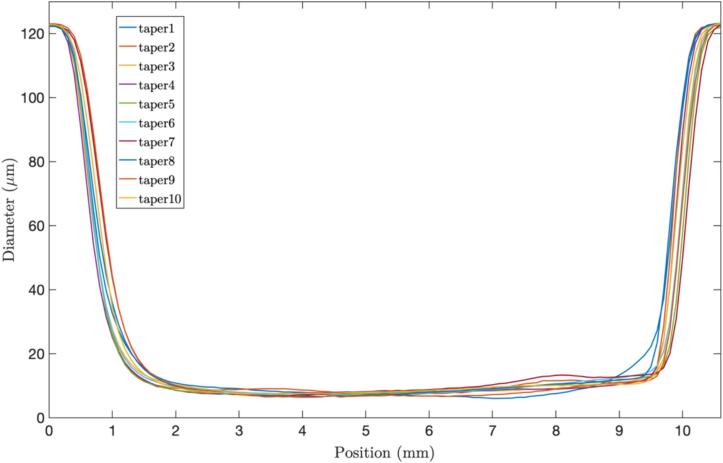
Taper profile of 10 tapers manufactured with 10 mm of pulling distance and 15 steps/s.

**Fig. 29:**
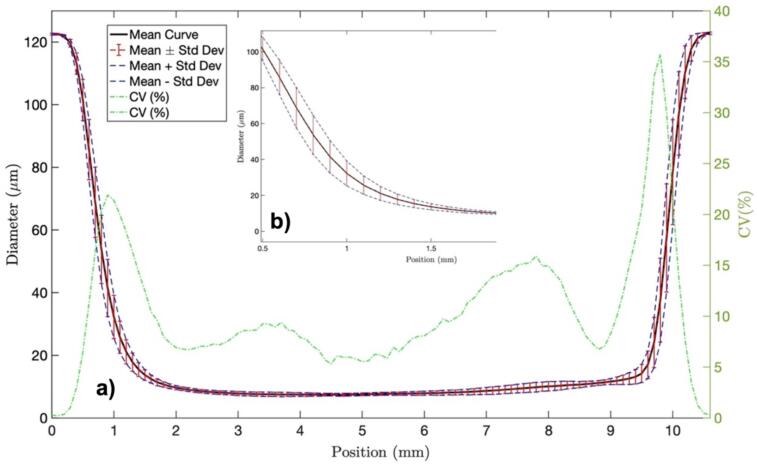
a) Mean curve, mean ± standard deviation and coefficient of variation. The repeatability of the manufacturing process is evaluated by deriving the main taper profile for the tapers and comparing the positive and negative standard deviations relative to the central curve as shown in Fig. 29b) The Coefficient of Variation (CV) is then calculated as the ratio of the standard deviation to the mean, representing repeatability between diameters at distinct positions.

In Fig. 28, we show the Taper Profile curve of 10 manufactured tapers, where it is evident that their dimensions are consistently similar, and the shapes of the up-down transitions exhibit uniform behavior. Although the down transition appears slightly less abrupt than the upper transition, this effect can be attributed to the system performance; it is noteworthy that the specific shape of the taper chosen for the input/output of light does not impact the spectral response because it remains the same. Furthermore, the diameter of the waist can be considered highly consistent, although some fluctuations may be observed in specific tapers. This variability is attributed to external factors, such as strong air currents, which can impact the alignment of the bare fiber with plasma (Fig. 29).

This study reveals variations in the up-down transitions, as previously mentioned. However, the dimensions of length, waist obtained, and curve shape are remarkably similar. Additionally, a consistent diameter of the waist is demonstrated, demonstrating an impressive fabrication performance of the tapers with an important level of repeatability and a reliable spectral response for potential optical applications.

### 7.3 Light transmission

**Fig. 30:**
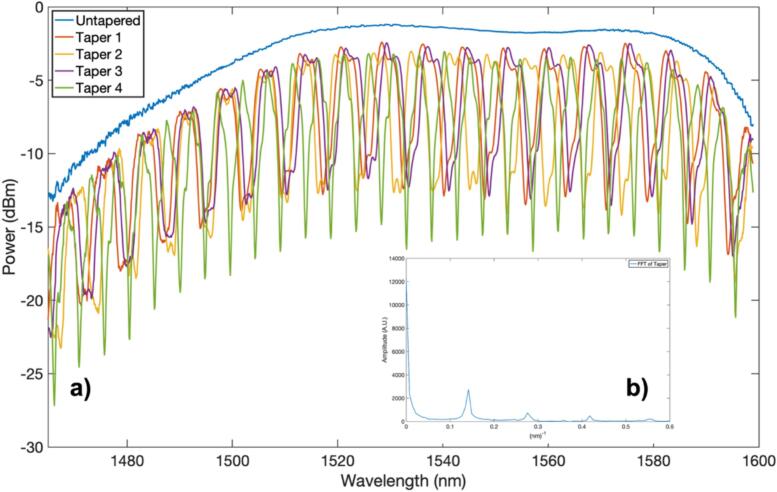
(a) Light transmission spectra of four 10-mm tapers with a diameter of 8 µm. The spectra show a Free Spectral Range (FSR) of 4.8 nm for the spectrum in green and 7.9 nm for the spectrum in red. (b) Fast Fourier Transform (FFT) of Taper 1, highlighting the fundamental mode and the excitation of higher-order modes. (For interpretation of the references to colour in this figure legend, the reader is referred to the web version of this article.)

To determine the losses and the light transmission of the tapers a Hewlett Packard HP8168C tunable laser operating within the wavelength range of 1465–1599 nm and a Hewlett Packard HP8153A Lightwave multimeter were employed. Light transmission through the fiber was measured both before and after the pulling process. This facilitated the setting of spectral references for both the untapered fiber referring to single-mode fiber (SMF), and the tapered fiber, as depicted in Fig. 30a).

The transmission of several tapers, characterized by the dimensions discussed earlier, was measured to estimate the typical optical response of the fabricated devices. Fig. 30(a) shows the spectrum of four of these devices, demonstrating consistency in spectrum shape. The visibility of the fringes ranges from 9 to 12 dBm, which is exceptional for amplitude optical response sensors. The average losses are less than 1.5 dB, considered low for these devices. Notably, the tapers exhibit an interference spectrum attributed to the excitation of higher-order modes, as observed in the Fast Fourier Transform (FFT) of the taper spectrum (see Fig. 30(b)). At least one higher mode is discernible, creating multimode interference with the fundamental mode.

## 8 Conclusions

A cost-effective, reliable optical fiber tapering system based on plasma as the heating source, was developed. The key features of the system: readily available hardware, open-source software and simple operation, makes it costly-effective and attractive for groups with an interest in applications based on tapered structures within a range of fields in science and engineering. The detailed description of the rig proves to be fundamental for other research groups to be able to reproduce it. Mechanically strong (compared to tapered devices manufactured via other techniques) tapers with interference fringes (with 12 dB depth) ranging from 1465 nm to 1599 nm, were obtained. The aforementioned characteristics makes them attractive for sensing, laser-based and broadband sources, and for optical communications applications. Full control engineering of important parameters such as: temperature gradient, differential pulling, and taper geometry will be carried out in the next iteration of the tapering system presented in this work.

## CRediT authorship contribution statement

**L.F. Granados-Zambrano:** Writing – original draft, Visualization, Software, Methodology, Conceptualization. **J.P. Korterik:** Writing – review & editing, Visualization, Validation, Supervision, Software, Resources, Methodology, Investigation, Conceptualization. **J.M. Estudillo-Ayala:** Supervision, Investigation. **R. Rojas Laguna:** Supervision, Investigation. **D. Jauregui-Vazquez:** Writing – review & editing. **H.L. Offerhaus:** Writing – review & editing, Project administration. **J.A. Alvarez-Chavez:** Writing – review & editing, Supervision, Conceptualization.

## Declaration of competing interest

The authors declare that they have no known competing financial interests or personal relationships that could have appeared to influence the work reported in this paper.
